# Emotion Regulation in Schizophrenia: A Pilot Clinical Intervention as Assessed by EEG and Optical Imaging (Functional Near-Infrared Spectroscopy)

**DOI:** 10.3389/fnhum.2018.00395

**Published:** 2018-10-09

**Authors:** Michela Balconi, Alessandra Frezza, Maria Elide Vanutelli

**Affiliations:** ^1^Department of Psychology, Catholic University of Milan, Milan, Italy; ^2^Research Unit in Affective and Social Neuroscience, Catholic University of Milan, Milan, Italy

**Keywords:** EEG, emotional behavior, fNIRS, neurofeedback, schizophrenia

## Abstract

Previous research on Schizophrenia (S) revealed anomalies in brain responsiveness during emotion processing, as shown by neuroimaging and electroencephalography (EEG) measures. Nonetheless preserved capacities to explicitly evaluate the emotional significance of affective stimuli in term of valence have been found. The present study applied functional Near-Infrared Spectroscopy (fNIRS) and EEG to explore the spatial and temporal expressions of emotion processing in the brain before (T0) and after (T2) an emotional Neurofeedback (NF) training of patients, assigned to the control or the experimental group. Explicit measures revealed correct identifications of stimuli emotional valence before (T0) and after (T2) the treatment, while implicit measures (EEG and fNIRS) showed a modulation and increased competencies only after the NF (T2), with more balanced prefrontal activity.

## Introduction

Schizophrenia (S) is a complex neuropsychiatric syndrome characterized by positive, negative and neurocognitive symptoms with enduring social impairment. Although positive symptoms usually respond to pharmacological treatment, negative symptoms tend to persist in the form of affective flattening, alogia, anhedonia, asociality and emotion dysregulation. In fact, affective deficits are considered pivotal in S since they affect patients’ personal and interpersonal spheres, and consequently their social functioning.

As pointed out by Balconi et al. (2015c) emotional deficits in S affect different processes, among which emotional experience (Taylor et al., 2012), face emotional expression (Blanchard and Cohen, 2006), as well as the domains of perception and recognition (Horan et al., 2011). Conversely, other emotional skills, such as the ability to explicitly evaluate the emotional valence of stimuli, appear relatively preserved (Kring and Moran, 2008). However, it has been suggested that results are often inconsistent. In fact, some evidence of affective impairment in S revealed that patients display “blunted” or “flat” facial and vocal affect, which was supposed to be related to diminished emotional experience. Nevertheless, in parallel, available data suggest that their subjective emotional experience is not consistently modified (Aghevli et al., 2003) and do not differ from healthy subjects with regard to valence and arousal ratings (Kring et al., 2003).

In recent times neuroimaging methods have been applied in support of the diagnostic process for different psychiatric disorders, to identify biological markers and the neural correlates underlying such pathologies (Linden and Fallgatter, 2009) by analyzing the recruitment of specific brain regions. Also, they began to be used to assess treatment effects, in a more objective way than conventional subjective outcome measures based on patients’ reports.

Neuroimaging techniques consist mainly of functional magnetic resonance imaging (fMRI), positron emission tomography (PET) and, more recently, near-infrared spectroscopy (NIRS). Such techniques, in fact, have already contributed to the knowledge of the pathophysiological features of mental disorders. Nonetheless, the clinical applications in treatment monitoring are still inconsistent. In fact, the brain effects of different psychiatric or psychological treatments still need to be explored, to reveal changes in information processing, to investigate the mechanisms of neural plasticity, and to compare the effects on the brain during different types of intervention, thus permitting the development of new interventions (Linden and Fallgatter, 2009; Balconi et al., 2018).

In the present study, functional NIRS (fNIRS) was used. fNIRS is a rather new technique in the clinical domain, that can quantify and measure the oxygenation level of brain tissues. Its use in psychiatry has been growing rapidly since it has better spatial resolution than electroencephalography (EEG) and better temporal resolution than fMRI. In fact, it is particularly suitable for clinical applications in that it can easily and non-invasively measure brain activity in a naturalistic position without movement limitation constraints. fNIRS instruments are also silent and portable: in fact, they can be moved almost everywhere. Also, fNIRS has a higher temporal resolution than fMRI or PET (Ferrari and Quaresima, 2012) and permits combined measurements with other neuroimaging techniques, such as EEG.

For what concerns the psychiatric field, Schizophrenia Spectrum Disorders was the most addressed topic by fNIRS research (Ehlis et al., 2014). One of the most significant findings from the previous evidence is that patients with chronic S display an abnormal activity in the frontopolar prefrontal cortex which proved to be associated with impaired functions (Koike et al., 2013), which are also core regions during emotion processing and regulation. As already suggested by Balconi et al. (2015c) in a recent review, one of the most interesting developments of fNIRS studies on schizophrenia is about emotional recognition, where this technique showed to be a valid tool to assess this functional impairment (Shoji et al., 2013).

Besides pathophysiological markers coming from neuroimaging research, some anomalies have been found in the electrocortical activity of patients with schizophrenia. For example, although results are still not always consistent, it seems that patients with schizophrenia show higher low-frequency electrocortical activity, with increased Delta and Theta bands. These low-frequencies bands are considered important in the motivational system and emotional processes: in fact, previous evidence suggested their involvement during the detection of the arousing power of stimuli and the motivational and attentional significance of relevant emotional cues (Balconi and Pozzoli, 2009). Also, the presence of abnormal lateralized prefrontal activity within the left or right hemisphere has been found (Gruzelier, 1999).

One possible application to manage such dysfunctional electrocortical response could be neurofeedback (NF), a technique that works eliciting desirable brain waves and inhibiting abnormal electrocortical activity (Kouijzer et al., 2009). It is based on the behavioral approach and operant conditioning paradigm and uses the shaping procedure (Sherlin et al., 2011) to heighten awareness, and reinforces voluntary control on electrophysiological components. The efficacy of NF on S has been demonstrated since the most preliminary study performed by Schneider et al. (1992), in which attention breakdown of patients with schizophrenia was ameliorated through regulating the slow cortical potentials. Also, in an in-depth case study, Schummer (2008) uncovered favorable effects of NF in a protracted process. The work of Surmeli et al. (2012), instead, is another example of NF treatments in cognition spheres of patients with schizophrenia. For what concerns interventions addressed to emotional impairment, Ruiz et al. (2013) trained nine patients with schizophrenia to up- and downregulate anterior insular cortex activity to improve face emotion recognition. A specific model that could also be followed for NF intervention is that of functional hemispheric imbalance, adopted, for example, by Gruzelier et al. (1999) who trained patients to learn interhemispheric control.

However, the existing studies are only preliminary and generally not exhaustive in term of experimental paradigm (no imaging evidences after NF treatment (Naimijoo et al., 2015), sample size (only single or few cases, Schummer, 2008; Balconi et al., 2018) or specific type of treatment).

Thus, starting from our previous protocol (Balconi et al., 2018) which was preliminarily applied to a small sample of patients, we conducted the present study with the aim to evaluate the efficacy of an NF intervention to improve emotional regulation in a larger sample of S patients by means of a neurophysiological assessment. The assessment was conducted during a passive emotional task to explore the brain mechanisms related to emotional processing before and after the training (pre/post), following a multi-method approach with both electrophysiological and hemodynamic measures. Indeed, despite the growing evidence demonstrating the efficacy of NF interventions according to specific pathophysiological indices, some caution points should be considered. As suggested by Hammond (2010), there is great heterogeneity of EEG patterns associated with various diagnoses and symptoms. Thus, the advantages of utilizing an objective EEG evaluation before NF treatment are in this case fundamental. Finally, together with the brain activity, the explicit subjective evaluation of emotional stimuli was recorded, to explore the relationship between the two compartments within a multi-method frame.

According to available evidence (Kikuchi et al., 2007), we hypothesized the prevalence of low-frequency bands (mainly Delta) over prefrontal regions during the emotional assessment. Also, we expected that patients’ electrocortical activity could be lateralized according to their specific symptomatology. In so far the NF intervention could be useful in balancing prefrontal Delta activity after the training, we expected a more “balanced” prefrontal response to the emotional cue, tested by both EEG and fNIRS measures.

## Materials and Methods

### Participants

The study recruited a pilot sample of 18 institutionalized patients, nine females and nine males (*M*_age_ = 34.12; SD = 7.09; range = 25–42). The sample is an extension of a preliminary data collection on nine patients (see Balconi et al., 2018). Establishment of diagnoses was based on semi-structured interviews which were conducted by an expert psychiatrist according to the criteria of the fourth edition of the diagnostic and statistical manual of mental disorders (Structured Clinical Interview for DSM IV Axis I Disorders, SCID-I).

This study was carried out in accordance with the recommendations of Ethical Committee of the Psychology Department of the Catholic University of Milan. The protocol was approved by Ethical Committee of the Psychology Department of the Catholic University of Milan. All subjects gave written informed consent in accordance with the Declaration of Helsinki. Inclusion criteria were: (1) age between 18 and 45 years old; (2) fair psychopathological compensation; and (3) stable pharmacological program followed for at least 4 weeks before the beginning of the study. Exclusion criteria were as follows: (I) visual or auditory impairment; (II) concurrent substance abuse (abstinence for at least 3 months); (III) diagnosis of moderate or severe mental retardation (IQ<55); (IV) neurological damage, including any neurological impairment (cerebrovascular or degenerative disorders), as investigated through patients’ medical history; and (V) anamnesis of brain injury.

Then, patients have been randomly assigned to either the control group (C), composed by nine patients, five females (*M*_age_ = 37; SD = 5.87; range = 27–41), or the NF group (N), composed by nine patients, four females (*M*_age_ = 35.98; SD = 6.55; range = 25–42). The two groups were comparable in term of emotional intelligence (Mayer-Salovey-Caruso Emotional Intelligence Test, MSCEIT), cognitive competencies (Wechsler Adult Intelligence Scale Fourth Edition, WAIS-IV) and neuropsychological profile (brief assessment of cognition in Schizophrenia, BACS). Patients within the C group simply followed the Treatment As Usual (TAU), while patients in the experimental group also participated in the NF training. A similar interaction (in term of number and quality of interaction) was applied to the control and experimental group.

The study included the first phase of assessment (T0), the NF intervention (T1), and a second assessment (T2) to evaluate the treatment efficacy. Procedures in T0 and T2 were identical to allow direct comparison of their measures and groups. The assessment involves the acquisition of neurophysiological parameters (both electrocortical and hemodynamic) by means of fNIRS and EEG while emotional pictures are presented. Moreover, the subjective ratings were also reported in terms of valence and arousal of stimuli, by means of the Self-Assessment Manikin (SAM; Russell, 2003).

### Stimuli

Patients were asked to look at the monitor where different affective patterns were displayed. Then, they were required to evaluate them in terms of valence and arousal at the end of the presentation. One hundred pictures were taken from the International Affective Picture System (IAPS; Bradley and Lang, 2007), depicting 40 positive and 40 negative pictures (20 low and 20 high arousing, each), and 20 neutral stimuli, based on valence and arousal ratings obtained from a previous study (Balconi et al., 2009). IAPS subjective ratings were obtained with the SAM scale, using an easier adapted 5-point version (Bradley and Lang, 2007). For IAPS codes see Balconi et al. (2015b).

### Procedure

**Figure 1:**
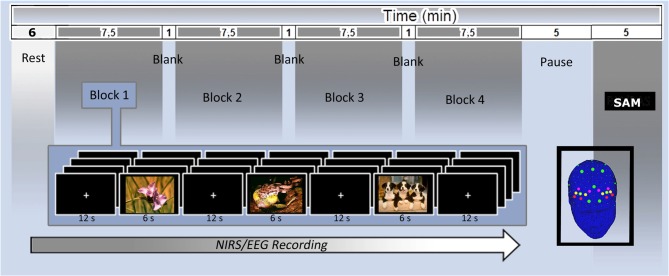
Electroencephalography (EEG; green) and functional near-infrared spectroscopy (fNIRS; yellow dots) montage and experimental setting with central and Self-Assessment Manikin (SAM) rating measures.

Patients were seated in a dimly lit room, in front of a computer monitor placed at a distance of 70 cm. The stimuli were presented with STIM software (Stim2, Compumedics Neuroscan, Charlotte, NC, USA) running on a 15-inch. screen. Participants were asked to observe each stimulus during the EEG/fNIRS recording for the entire time of exposition. Pictures were presented randomly at the center of a computer monitor for 6 s, with an inter-stimulus interval of 12 s. One-hundred and twenty seconds eyes-closed and 120 eyes-open resting baseline were registered at the beginning of the experiment before picture presentation. After the experimental phase, patients had time to rate their emotional experience on the SAM scale (see Figure 1).

### EEG Recording and Analysis

To implement a personalized training according to each patient’s specific features, in parallel with fNIRS recording, electrophysiological measures were acquired. A 16-channel portable EEG-System (V-AMP: Brain Products, München) was used for data acquisition. An ElectroCap NIRS-EEG compatible with Ag/AgCl electrodes was applied to record EEG from active scalp sites referred to earlobe (10/5 system of electrode placement; Oostenveld and Praamstra, 2001). EEG activity was recorded on positions AFF3, AFF4, Fz, AFp1, AFp2, C3, C4, Cz, P3, P4, Pz, T7, T8, O1, O2 (see Figure 1). Additionally, one EOG electrode was placed on the outer canthi to detect eye movement. The data were recorded using a sampling rate of 500 Hz, with a notch filter of 50 Hz. The impedance of recording electrodes was monitored for each subject prior to data collection and it was always kept below 5 kΩ. Blinks were also visually monitored. Ocular artifacts (eye movements and blinks) were corrected using an eye-movement correction algorithm that applies a regression analysis in combination with artifacts averaging. After EOG correction and visual inspection, only artifact-free trials were considered. To obtain a signal proportion to the power of the EEG frequency band, the filtered signal samples were squared and successively log-transformed (Pfurtscheller, 1992). Successively, the data were epoched, using time window of 1,000 ms and an average absolute power value was calculated for each electrode, for each condition. Artifact-free data have been used to compute power spectra for relevant EEG frequency bands by Fast Fourier transform method (Hamming window: length 10%) that was used to obtain estimates of spectral power (μV^2^) in the 1 Hz frequency bins for each electrode site. Spectral power values were averaged across all epochs and were then transformed to power density values for the different frequency band. Digital EEG data (from all 15 active channels) were band-pass filtered in the following frequency bands: Delta (0–3), Theta (4–7), Alpha (8–12), Beta (13–20). During data reduction, a bandpass filter was applied in the 0.01–50 Hz frequency band.

### fNIRS Recording and Analysis

fNIRS measurements were conducted with NIRScout System (NIRx Medical Technologies, LLC., Los Angeles, CA, USA) using a 6-channel array of optodes (four light sources/emitters and four detectors) covering the prefrontal area. Emitters were placed on positions AF3-AF4 and F5-F6, while detectors were placed on AFF1-AFF2 and F3-F4 (see Figure 1). Emitter-detector distance was 30 mm for contiguous optodes and a near-infrared light of two wavelengths (760 and 850 nm) was used. With NIRStar acquisition software, changes in the concentration of oxygenated (O_2_Hb) and deoxygenated hemoglobin (HHb) were recorded continuously throughout the paradigm. Signals obtained from the six NIRS channels were measured with a sampling rate of 6.25 Hz, analyzed and transformed with nirsLAB software (v2014.05; NIRx Medical Technologies LLC, 15Cherry Lane, GlenHead, NY, USA), according to their wavelength and location, resulting in values for the changes in the concentration of O_2_Hb and HHb for each channel. Such values were obtained by applying the Beer-Lambert Law with Grazer spectrum to obtain the molar extinction coefficients of oxy and deoxy corresponding to wavelengths of 760 and 850 nm, respectively. The raw data of O_2_Hb and HHb from each channel were digitally band-pass filtered at 0.01–0.3 Hz. Then, the mean concentration of individual channel was calculated by averaging data across trials from the trial onset for 6 s. The mean concentration value of 6 s immediately before each trial was used as an event-related baseline. Based on the mean concentrations in the time series, we calculated the effect size in every condition for each channel within a subject, calculated as the difference of the means of the baseline (m1) and trial (m2) divided by the standard deviation (s) of the baseline: d = (m1 − m2)/s. Then, the effect sizes obtained from the six channels were averaged in order to increase the signal-to-noise ratio. These normalized effect sizes could be averaged regardless of the unit (for this procedure see Balconi et al., 2015a,b,d). To interpret the event-related responses to stimuli with respect to each baseline, signs have been inverted.

### Neurofeedback Training (T1)

The training consisted of a 5-weeks successive training period. The experimental group completed 10 sessions, each lasting approximately 25-min with two intervals of 2 min in between. The sessions were performed in the same room as the assessment phases. NF was administered using a Procomp2 device (Thought Technology Ltd., Montreal West, QC, Canada) and the task was created and presented by BioGraph Infiniti software package (Thought Technology Ltd., Montreal West, QC, Canada). Before each training session, a 2-min resting baseline was recorded with eyes open. Patients were instructed to reduce muscle activity and control eye blinks. EEG was recorded by placing an electrode in correspondence to the left (F3) or the right DLPFC (F4), according to each patient’s electrophysiological pattern. The reference was placed on the contralateral earlobe and the ground electrode on the ipsilateral earlobe. The main visual feedback and reward tool was a video made up of IAPS pictures different from those used during the initial assessment phase: when the EEG band of interest reached values over the established reward-threshold, the video proceeded and showed different affective stimuli. The reward threshold is automatically managed by the software such that a fixed 80%-reward level is provided. The band values were as follows: reward = 0.5–5.5 Hz; inhibit-low = 0.1–0.5 Hz; inhibit-high = 50–64 Hz.

## Results

### SAM Ratings

Arousal and valence subjective scorings (dependent measures) were analyzed with two separated three repeated factor (2 arousal; 3 valence; 2 time) and one between factor (C group vs. N group) mixed-model ANOVAs. For all ANOVA tests, degrees of freedom were corrected by Greenhouse–Geisser epsilon where appropriate. Moreover, due to multiple independent analyses and comparisons, we applied Bonferroni test for inequality. Contrast analyses (paired comparisons) were applied to significant main or interactions effects.

For valence ratings, Valence main effect was significant (*F*_(2,35)_ = 8.13, *p* < 0.01). Indeed negative valenced stimuli received significantly (*p* < 0.01, *η*^2^ = 0.32) lower values (*M* = 1.88; SD = 0.11) than positive stimuli (*M* = 4.77; SD = 0.54), with intermediate level for neutral ones (*M* = 2.94; SD = 0.2), which were significantly higher (*p* < 0.01, *η*^2^ = 0.34) than negative, and lower (*p* < 0.5, *η*^2^ = 0.28) than positive pictures. Also, Time main effect was significant (*F*_(1,6)_ = 7.45, *p* < 0.01, *η*^2^ = 0.33), with increased values for all conditions in T2 (*M* = 3.77; SD = 0.18) than T0 (*M* = 3.02; SD = 09). Finally, interaction effect Valence *Time* Group was significant, with negative pictures evaluated as more positive (*p* < 0.05, *η*^2^ = 0.29) by N group in T2 (*M* = 2.88; SD = 0.13) than T0 (*M* = 1.65; SD = 0.12). In contrast, C group did not show this significant effect. For arousal ratings, instead, no significant effect emerged.

### EEG Results

Repeated measure ANOVAs were applied to EEG band power, which have been averaged within each group for a direct comparison. Preliminary results showed, for all patients (both C and N) higher values for Delta band (*M* = 1.67; SD = 0.75) than Theta (*M* = 0.55; SD = 0.35), Beta (*M* = 0.18; SD = 0.07) and Alpha (*M* = 0.61; SD = 0.34; mean *η*^2^ = 0.32). Then, considering only Delta band, further analyses were conducted to explore which brain regions showed the highest effect. Results revealed that Frontal regions exhibited the highest values (*M* = 1.81; SD = 1.49) compared to Temporo-Central (*M* = 1.09; SD = 0.41) and Parietal ones (*M* = 1.28; SD = 0.45; mean *η*^2^ = 0.31).

Successively, for each patient a lateralization index was calculated to assess frontal brain asymmetry, as follows: (R − L)/(R + L). R and L stand for Delta activity over right (R) or left (L) hemisphere. Consequently, a frontal setting was considered within the range of Delta band, and on the hemisphere with the lower power, specific for each patient.

Then, the frontal brain log-transformed asymmetry (LTA) recorded in T0 and T2 was compared between patients in the N and C group to verify the efficacy of the functional hemispheric balancing. Positive values indicate right-sided patterns, while negative values indicate left-sided patterns. Values around zero indicate a general balance. Significant findings revealed that Delta activity was rather balanced after the treatment between the two hemispheres for N group (*M* = −0.04; SD = 0.09, no significant difference between left/right) while before the treatment it was, on average, more right-distributed (*M* = 0.13; SD = 0.03, *η*^2^ = 0.32). In contrast, C group showed similar EEG profile in T0 and T2 (no significant difference between T0/T2, p > 0.05, *η*^2^ = 0.28), with higher right-distributed values for both T0 (*M* = 0.19; SD = 0.04) and T2 (*M* = 0.21; SD = 0.02).

### fNIRS Results

Also, in the case of fNIRS recordings, repeated measure ANOVAs were applied with respect to D dependent measure of O_2_Hb concentration. A preliminary analysis did not reveal significant effects for HHb measure. Therefore, we reported only results for O_2_Hb.

Data were averaged considering left (Ch1: AF3-F3; Ch2: AF3-AFF1; Ch3: F5-F3) and right (Ch4: AF4-F4; Ch5: AF4-AFF2; Ch6: F6-F4) regions to obtain an inclusive index based on the specific lateralized NF application. Results showed that the N group presented different O_2_Hb levels in T0 and T2 (*p* < 0.01, *η*^2^ = 0.32), with more right hemodynamic concentrations in T0 (*M* = 0.23; SD = 0.09) and more balanced O_2_Hb levels in T2 (*M* = −0.03; SD = 0.11). In contrast C group showed higher right O_2_Hb levels in T2, after their sessions (*M* = 0.12; SD = 0.08), similarly to the more right O_2_Hb levels in T0 (*M* = 0.16; SD = 0.10; mean *η*^2^ = 0.30).

## Discussion

The aim of the present study was to provide a new neuroscientific-led method to assess and train emotion perception and regulation in patients with S. A multi-method approach was applied in order to record both subjective/explicit data (acquired through SAM questionnaire) and implicit/neural evidence (both hemodynamic and electrophysiological) before and after a NF intervention. Starting from previous evidence about the presence of dysfunctional emotional mechanisms with asymmetric neutral patterns, the protocol was meant to reestablish hemispheric unbalance in patients with S. In line with our preliminary results, the analyses revealed four major results of interest: (I) the capacity to assess emotional valence to the affective pictures was preserved in both groups of patients, while arousal evaluation revealed a difficulty in determining stimuli salience; (II) the initial assessment revealed the prevalence of Delta waves in patients’ cortical activity during emotional processing; and (III) the NF training could restore a balanced interhemispheric state, regardless of the lateralization effect.

First, the attributional process was explored: for what concerns valence ratings, all patients were able to correctly discriminate between positive, negative and neutral stimuli, both in T0 and T2. Indeed, it is possible to argue that this capacity does not derive from the NF training. Nonetheless, after the training, negative stimuli received more positive values compared to T0: this effect, instead, could be due to the NF practice and a subsequent more functional management of negative affects (Balconi et al., 2015c), even if it should be tested in a successive specific future analyses for a complete comprehension. In parallel, patients encountered more difficulties in discriminating the arousing power of pictures, which could derive from an impairment in detecting the motivational significance of external stimuli (Williams et al., 2004).

Second, Delta band was found to be over-represented in patients’ cortical activity during the emotional task, as found in T0. This is particularly interesting considering that anomalies in low-frequencies bands are thought to be involved in the motivational system and emotional processes: in fact, they were found to be related to arousing power of stimuli and may be responsive to the motivational and attentional significance of relevant emotional cues (Balconi et al., 2015b).

Third, the hemispheric imbalance training had favorable effects as found by fNIRS and EEG. For what concerns imaging data, O_2_Hb levels were more balanced in N group in T2 compared to T0, thus suggesting a functional involvement of frontopolar prefrontal regions. Also, this effect was visible over both F3 and F4 considered together, thus suggesting a balancing effect of the treatment. For what concerns EEG, frontal Delta LTA was found to be more pronounced in T0 and more balanced in T2 as a result of the NF training. Indeed, irrespective of the lateralization, the training was associated with an enhanced cortical balanced activity, as revealed by O_2_Hb levels. Therefore, the NF effect was effective in modulating the cortical left/right prefrontal activity in relationship with the pre-treatment condition specific for each subject.

To conclude, as pointed out by the present results, the integration of fNIRS and the EEG measures may be considered a valid method to be used to test the efficacy of clinical treatment (NF) by contemporarily elucidating the cortical oscillation and the hemodynamic effects obtained in PFC.

However, future developments could better consider the lateralization effect in response to specific emotional valence: indeed, as shown by previous studies, the left/right activity is related respectively to the positive vs. negative content of the emotional cues, with possible effects on the successive lateralized “balancing effect.” Future research should also point out the role of other frequency bands in addition to Delta band, to explore an ampler range of frequencies in relation to the emotional behavior. In addition, the same paradigm could be extended to even larger samples to overcome the limitation of small samples.

Finally, the clinical assessment should guide the final evaluation of the NF treatment effect, since the long-lasting evaluation of the emotional deficits should confirm the positive impact found for both EEG and fNIRS profile. Accordingly, some other important factors, such as disease duration and comorbidity could be properly taken into account. Moreover, the presence of an expert professional to guide the NF intervention could make up for the experimental setting involving some limitations to an ecological assessment, such as, for example, the technical problems of the different devices, the repetitive effect of the training, the mistrust to the complex technological apparatus, and so on.

## Author Contributions

MB planned the experiment, supervised the experiment, statistical analysis and wrote the article. AF executed the experiment, realized the statistical report and wrote the methodological part of the article. MV executed the experiment, realized the statistical analysis and wrote the article.

## Conflict of Interest Statement

The authors declare that the research was conducted in the absence of any commercial or financial relationships that could be construed as a potential conflict of interest.
